# Field Verification of an African Swine Fever Virus Loop-Mediated Isothermal Amplification (LAMP) Assay during an Outbreak in Timor-Leste

**DOI:** 10.3390/v12121444

**Published:** 2020-12-15

**Authors:** Peter T. Mee, Shani Wong, Kim J. O’Riley, Felisiano da Conceição, Joanita Bendita da Costa Jong, Dianne E. Phillips, Brendan C. Rodoni, Grant T. Rawlin, Stacey E. Lynch

**Affiliations:** 1 Agriculture Victoria Research, AgriBio Centre for AgriBioscience, Bundoora, VIC 3083, Australia; shani.wong@agriculture.vic.gov.a (S.W.); Kim.ORiley@agriculture.vic.gov.au (K.J.O.); Brendan.Rodoni@agriculture.vic.gov.au (B.C.R.); grant.rawlin@agriculture.vic.gov.au (G.T.R.); stacey.lynch@agriculture.vic.gov.au (S.E.L.); 2 Ministry of Agriculture and Fisheries, Government of Timor-Leste, Av. Nicolao Lobato, Comoro, Dili 0332, Timor-Leste; felisianodaconceicao@gmail.com (F.d.C.); katitadog_2001@yahoo.com (J.B.d.C.J.); 3 Agriculture Victoria, Biosecurity and Agriculture Services, Bairnsdale, VIC 3857, Australia; dianne.phillips@agriculture.vic.gov.au

**Keywords:** ASFV, LAMP, qPCR, colourmetric LAMP, Timor-Leste

## Abstract

Recent outbreaks of African swine fever virus (ASFV) have seen the movement of this virus into multiple new regions with devastating impact. Many of these outbreaks are occurring in remote, or resource-limited areas, that do not have access to molecular laboratories. Loop-mediated isothermal amplification (LAMP) is a rapid point of care test that can overcome a range of inhibitors. We outline further development of a real-time ASFV LAMP, including field verification during an outbreak in Timor-Leste. To increase field applicability, the extraction step was removed and an internal amplification control (IAC) was implemented. Assay performance was assessed in six different sample matrices and verified for a range of clinical samples. A LAMP detection limit of 400 copies/rxn was determined based on synthetic positive control spikes. A colourmetric LAMP assay was also assessed on serum samples. Comparison of the LAMP assay to a quantitative polymerase chain reaction (qPCR) was performed on clinical ASFV samples, using both serum and oral/rectal swabs, with a substantial level of agreement observed. The further verification of the ASFV LAMP assay, removal of extraction step, implementation of an IAC and the assessment of a range of sample matrix, further support the use of this assay for rapid in-field detection of ASFV.

## 1. Introduction

African swine fever (ASF) is a highly contagious, viral disease in swine characterised by fever, haemorrhages and a high mortality rate [1]. The disease is caused by African swine fever virus (ASFV), the only member of the genus *Asfivirus* family Asfarviridae [1]. The transboundary nature of the disease saw outbreaks from Africa into Eastern Europe in 2007 (genotype II, Georgia 2007) [2] and has since spread throughout the world. Recently, the continued movement of the virus saw large outbreaks in China in August 2018 [3] and other Asian countries such as Vietnam [4], Cambodia, the Philippines [5] and Korea [6] in 2019. Once established in a region, the virus can rapidly spread throughout a swine population. After infection, death can occur in 2–10 days, with mortality rates seen as high as 100% [7]. Currently, there is no licenced vaccine available for the prevention of ASF. As a result, rapid diagnosis and the implementation of movement controls are the most effective methods to limit spread of the virus [8]. Complicating diagnosis in many locations is the cocirculation of other swine diseases. Diseases such as classical swine fever (CSF), porcine dermatitis and nephropathy syndrome (PDNS) and porcine reproductive and respiratory syndrome (PRRS) have similar clinical presentation, making the true extent of the ASF outbreaks difficult to assess [9,10,11]. Many locations that these outbreaks have occurred are in remote, and often resource-limited countries without definitive tests for rapid diagnosis [12].

The transmission of ASFV is primarily through direct contact of swine with infected swine [13]. It can occur through exposure to infected blood, saliva, urine and faeces [13,14], and the consumption of contaminated feed and pork products [15]. The virus is very resistant to environmental and physical factors and can remain viable in the environment for an extended period [16]. ASFV can spread through insect vectors, with soft tick species in the genus *Ornithodoros* acting as a natural reservoir in some countries [17]. Experimentally, *Stomoxys* flies (Diptera: Muscidae) and swine lice, *Haematopinus suis* (Phthiraptera: Haematopinidae) have also been shown capable of transmitting ASFV via mechanical mechanisms [18,19].

In September of 2019, following large pig mortalities, ASF was first detected in Timor-Leste [12]. Discovered in the capital city, Dili, the initial outbreak had 405 cases and a case fatality rate reported of 100% [20]. Pigs are kept by over 70% of households in Timor-Leste and hold a significant monetary and cultural value within the economy of ceremonies [12]. The current extent of the outbreak in Timor-Leste is currently unknown due to limited testing facilities.

Recent years have seen a rapid expansion in a range of technologies developed for the detection of ASFV and specific antibodies. This has included the development of indirect ELISAs for the detection of ASFV antibodies in serum and oral fluids [21], which can be sensitive but time consuming (2 h), are not field applicable nor able to detect a virus in clinical material. Nucleic acid based assays such as clustered regularly interspaced short palindromic repeats (CRISPR)/Cas12a technology has also been applied to ASFV diagnostics to develop detection methods that are both instrument-free through lateral flow devices [22] or in conjunction with point of care devices such as benchtop fluorescence-sensing units [23]. These developments have progressed the field applicability of ASFV diagnostics but still require the release of the genomic DNA through treatment with lysis and stabilizing buffers, before a recombinase-aided amplification (RAA) and CRISPR/Cas12a—lateral flow detection, which can take 1 h [22] or up to 2 h [23]. Although many advances have been made in detection sensitivity, specificity and turnaround time to results for ASFV diagnostic there is still a need to further develop techniques that can truly be used in a field situation.

The use of loop-mediated isothermal amplification (LAMP) as a nucleic acid amplification technique is increasing in its position to rapidly test samples for a range of organisms in a pen-side, point-of-care or laboratory setting [24]. LAMP reactions consist of a set of four to six primer pairs, which increase the assay specificity and reduce detection times [24,25]. The *Bst* DNA polymerases used in LAMP assays are robust, less affected by PCR inhibitors and can be used with unextracted nucleic acid resulting in an overall more efficient and cost-effective test than quantitative polymerase chain reaction (qPCR) [26,27,28]. Additionally, as LAMP reactions occur at a single amplification temperature, the need for expensive thermal cycling machines is negated [24,25]. A series of different detection formats have been developed for visualizing LAMP results from running LAMP products on agarose gels [24]; endpoint detection through colour change or development of turbidity [29]; or real-time analysis through the detection of intercalating dyes [30]. Recent studies have demonstrated that LAMP can have comparable specificity and sensitivity to other nucleic acid amplification techniques such as polymerase chain reaction (PCR) and qPCR [31,32,33,34]. With the identification of new application for isothermal enzymes, emerging methods such as cross-priming amplification (CPA) assays are being developed utilizing similar properties to LAMP [35].

Several LAMP assays exist for the detection of ASFV, with preliminary validation of these assays performed on extracted nucleic acid [36,37,38]. The LAMP assay targeting the topoisomerase II (TPII) gene developed at the Institute for Animal Health, United Kingdom, was selected for further in-field verification as part of our study [36]. This published assay demonstrates a high degree of specificity for ASFV, detecting all circulating genotypes without cross-reaction to classical swine fever virus (CSFV) [36]. The assay has previously shown a “good” level of agreement with qPCR when testing extracted nucleic acid from clinically positive material [36]. Further verification is required to validate the assays when using unextracted or “raw” clinical samples, which would be processed in a field or outbreak situation. Whole blood is one sample type that is likely to be used due to the high levels of ASFV that can be detected in the blood during infection [14]. However, there is limited knowledge if anticoagulants used to collect clinical samples such as whole blood, can delay LAMP pathogen detection times or inhibit reactions as has been observed when using molecular assays such as PCR [39,40]. Additionally, there is limited knowledge if dry blood swabs, which are commonly used for the detection of ASFV, would interfere with the LAMP assays ability to detect this pathogen.

The development and distribution of simple to use portable instruments such as the Genie III system by OptiGene has enabled LAMP technology to be successfully utilized to diagnose infectious diseases in a remote field and resource-limited situations [34]. In this study, we report the verification of a LAMP assay to detect ASFV directly from clinical samples collected during an outbreak in Timor-Leste. Furthermore, we assess the performance of the test across several relevant sample matrices (including whole blood and clinical swabs) and investigate the ability of colourmetric LAMP reagents. In-field verification of the assay was cross-validated in Timor-Leste using an Office International des Epizooties (OIE) recommended ASFV qPCR assay [41], deployed on a hand-held, portable Biomeme qPCR machine.

## 2. Materials and Methods

### 2.1. Preparation of a Synthetic DNA Positive Control and Modified Internal Amplification Control

A dsDNA gBlock Gene Fragment (Integrated DNA Technologies Inc., Coralville, IA, USA) was synthesised as a positive control for ASFV and initial primer optimization. The gBlock was made by aligning the two outer LAMP primers (F3/B3) [36] (Table 1) to Malawi Lil-20/1 ASFV genome (GenBank accession AY261361), covering a 206 bp region of the TPII (Table 2). The gBlock was amplified using 1 µL of each F3 and B3 ASFV LAMP primer at 10 µM using Platinum^TM^ PCR SuperMix High Fidelity (Invitrogen, Carlsbad, CA, USA) following the manufacturer’s protocol. The amplified product was purified using an Isolate II PCR and Gel Kit (Bioline, Taunton, MA, USA) and quantified using dsDNA HS Assay Kit on a Qubit^TM^ 2.0 (Invitrogen Carlsbad, CA, USA) fluorometer. A second gBlock was designed to be used as an exogenous internal amplification control (IAC). Briefly, the IAC gBlock targeted the same region as the ASFV gBlock apart from the addition of 8 nucleotides (Table 2) to elevate the annealing temperature. The IAC gBlock was prepared as outlined above.

### 2.2. ASFV LAMP

The LAMP primers for the ASFV assay targeting the TPII gene were obtained from James et al. 2010 (Table 1). The LAMP assay parameters were optimised for the amplification of nucleic acid directly from clinical samples, without prior DNA extraction. LAMP reactions were performed using 15 µL of Isothermal Mastermix ISO-DR004 (OptiGene Ltd., Horsham, UK), 2.5 µL of primer mix with a final concentration of F3/B3 0.2 µM, FIP/BIP 1.6 µM and loop primers at 0.8 µM (Sigma-Aldrich, Castle Hill, Australia), 2 µL of template, with nuclease-free water making the reaction up to 25 µL. All samples were tested in parallel for inhibition using the IAC gBlock in separate wells to ensure no competition with ASFV detections occurred. Reactions were performed as outlined above, apart from the addition of 2 µL of the IAC gBlock.

LAMP reactions were performed on a Genie III (OptiGene, Horsham, UK) instrument with run conditions of 65 °C for 25 min, with annealing performed from 98 to 80 °C ramping at 0.05 °C per second. Results from the Genie III instrument will be reported here as the time to positive (T_P_) (minutes: seconds), and the anneal derivative temperature (T_a_; °C). All LAMP analysis was performed using Genie^®^ Explorer v2.0.6.3 software (OptiGene, Horsham, UK) using default thresholds. Samples were called positive for ASFV if an amplified product had an average T_a_ of 87.42 °C (± 0.56 °C) and the T_p_ < 20 min. A sample was deemed not affected by inhibitors if the IAC gBlock was detected with a T_a_ of 89.5 °C (± 0.4 °C) and a T_p_ of 10:39 (± 4:00).

### 2.3. Sample Matrix Assessment

A series of sample collection matrices were tested to determine the optimal matrix for screening using the ASFV LAMP assay. Five different matrices and water were tested including, viral transport medium (VTM; brain–heart infusion (BHI) broth (OXOID) containing 2 × 106 U/L penicillin (Seqirus, Melbourne, Australia), 0.2 mg/mL streptomycin (Sigma Aldrich, Stockholm, Sweden), 0.5 mg/mL gentamicin (Sigma Aldrich) and 500 U/mL amphotericin B (Sigma Aldrich)), porcine serum, whole porcine blood collected in fluoride oxalate tubes (BD Vacutainer^®^), EDTA tubes (BD Vacutainer^®^) and lithium heparin tubes (BD Vacutainer^®^). To test if the sample matrix would affect the detection of the ASFV LAMP, the sample matrices were tested at a neat, 1 in 10 and 1 in 100 dilution in nuclease-free water before being spiked with ASFV gBlock. The ASFV gBlock spikes were performed at a low and medium level equating to 200 and 20,000 copies/µL, respectively. Samples were tested in triplicates, with 2 µL used for each reaction.

### 2.4. Blood Swab Assessment

Dry blood swabs were assessed for their ability to be used with the ASFV LAMP assay in comparison to fresh blood swabs. Whole porcine (*Sus scrofa domestica*) blood collected in EDTA tubes (BD Vacutainer^®^) was spiked with a low (200 copes/µL) concentration of the ASFV gBlock and a medium (20,000 copies/µL) concentration. Three 15 cm CLASSIQSwabs (Copan) were dipped into each concentration and allowed to dry overnight at room temperature. Additionally, three swabs at each concentration were also dipped and tested fresh on the LAMP assay. Before testing, swabs were placed in 500 µL of nuclease-free water for a period of 10 min with intermittent stirring to elute blood off the swabs. Next 2 µL of the sample was heat-treated in the Genie III machine at 95 °C for 2 min, before testing using the ASFV LAMP assay in triplicates.

### 2.5. Analytical Specificity

The analytical specificity of the ASFV LAMP was assessed for potential off-target interactions with the sample matrix of whole blood. To evaluate this a 160 whole porcine (*Sus scrofa domestica*) blood samples were collected in fluoride oxalate tubes (BD Vacutainer^®^) from an ASFV free area (an abattoir in Victoria, Australia) and store at 4 °C for up to two weeks before testing. Samples were mixed via inversion several times before diluting 1 in 10 in nuclease-free water with 2 µL used for the ASFV LAMP assay.

### 2.6. Repeatability

The repeatability of the ASFV LAMP assay was assessed using the sample matrices of whole porcine blood that had been collected in fluoride oxalate tubes (BD Vacutainer^®^) and porcine serum. Both sample matrices were diluted 1 in 10 in nuclease-free water before being spiked with a low (200 copies/µL) and medium (20,000 copies/µL) ASFV gBlock. Samples were vortexed before 2 µL was transferred into a LAMP tube and heat-treated at 95 °C for 2 min, then tested using the ASFV LAMP assay. Intra-assay variability was assessed by performing six replicates within each run, and interassay variability was evaluated by repeating three independent runs. The coefficient of variation (CV) was used to assess the variability of the assay by measuring the T_p_ of the ASFV gBlock at different concentrations.

### 2.7. Limit of Detection

Limit of detection of the ASFV LAMP assay was performed in three sample matrices; nuclease-free water, whole porcine blood that had been collected in fluoride oxalate tubes (BD Vacutainer^®^) and porcine serum. The matrices of serum and blood were diluted 1 in 10 in nuclease-free water, before all three matrices were spiked with the ASFV gBlock. A ten-fold serial dilution of the gBlock in nuclease-free water was performed with 5 µL of gBlock spiked into the 45 µL of the diluted sample matrix, resulting in 2 × 10^7^–2 × 10^1^ copies/µL being tested. Two microliters of each dilution was tested in triplicates for each of the three sample matrices.

### 2.8. Colourmetric LAMP

Colourmetric LAMP reactions were performed with 12.5 µL of WarmStart^®^ Colorimetric LAMP 2X Master Mix (New England BioLabs, Ipswich, MA, USA), 2.5 µL of ASFV LAMP primer mix at the concentration outlined above, 2 µL of sample and 8 µL of nuclease-free water per reaction. Samples were incubated in a 65 °C water bath for 30 min before checking the assay for the observed colour change. A positive was classified on a colour change from pink to yellow, with an orange colour classified as an indeterminate result.

### 2.9. Sample Collections in Timor-Leste

Clinical samples were collected in September 2019 from 37 local pigs (*Sus celebresis timoriensis*) around the municipality of Baucau (8.4731° S, 126.4554° E) the second-largest city in Timor-Leste. Pigs presented with clinical signs consistent with ASF; including loss of appetite, vasculitis, bloody diarrhoea and vomiting. Blood was collected from the caudal aspect of the axilla via a subclavian venupuncture using serum tubes (BD Vacutainer^®^). Samples were transported on ice back to the laboratory. Upon receipt into the laboratory, serum was separated by spinning the blood tubes at 3000× *g* for 20 min. Serum was transferred to a 5 mL tube before being stored at −20 °C until testing. 15 cm CLASSIQSwabs (Copan) were used to collect oral and rectal swabs, which were combined and stored in 2 mL of phosphate buffer solution (PBS) at 4 °C for up to 1 month before testing.

### 2.10. ASFV LAMP Sample Preparation in Timor-Leste

Serum, blood and swab samples tested using either the ASFV LAMP and colourmetric ASFV LAMP assay were first diluted using a 10 µL disposable loop (Copan) into 90 µL of nuclease-free water. Samples were homogenized by flick mixing, and 2 µL of samples was transferred into a LAMP strip tube. Samples were heat-denatured at 95 °C for 2 min in the Genie III machine before cooling the sample to room temperature. Due to a lack of DNA extraction, and as ASFV is enveloped, heat shocking was included to denature the viral capsid, exposing viral DNA and inactive the virus [42].

### 2.11. ASF qPCR Assay in Timor-Leste

To cross-verify the ASFV LAMP results, all 37 pig samples from Baucau were tested using an OIE recommended ASFV qPCR assay [9]. DNA was extracted from 500 µL of serum with the M1 Bulk Sample Prep Kit for DNA (Biomeme, Philadelphia, PA, USA), as per manufacturer’s instructions apart from a final elution in 200 µL of the Biomeme elution buffer. Samples were run on the two3 (Biomeme, Philadelphia, PA, USA), portable qPCR machine, which allowed for three reactions at a time, consisting of a sample, a synthetic positive control and no template negative control per run. The synthetic positive qPCR control was generated using DNA from a gamma-irradiated ASFV genotype II. Briefly, the DNA was extracted using a DNeasy Blood and Tissue Kit (Qiagen, Valencia, CA, USA) as per the manufacturer’s instructions; the sample was amplified using 1 µL of each 10 µM primer from the King et al. 2003 assay with Platinum^TM^ PCR SuperMix High Fidelity (Invitrogen, Carlsbad, CA, USA) following the manufacturer’s protocol. The amplified product was purified using an Isolate II PCR and Gel Kit (Bioline, Taunton, MA, USA), before performing a 1 in 100,000 dilution in nuclease-free water to generate a working stock. qPCR reactions were performed in empty Go-Strips (Biomeme, Philadelphia, PA, USA). Reactions were set up using the AgPath-ID^TM^ One-Step RT-PCR kit (Applied Biosystems, Foster City, CA, USA) with 7.5 µL of 2 × AgPath RT-PCR Buffer, 1 µL of 25 × AgPath RT-PCR Enzyme Mix, 0.9 µL of primer probe mix (with primers at a final concentration of 300 nM and probe at 250 nM), 5 µL of the template and 0.6 µL of nuclease-free water. Reaction were performed with an initial denaturation/activation at 95 °C for 10 min, followed by 40 cycles of 95 °C for 15 s and 60 °C for 45 s. A positive result was determined, if nothing was detected in the negative control, the positive control had a Cq value of 11.5 (± 2.5) and the sample had a Cq value below 40.

### 2.12. Ethics

No ethical approval was required as no clinical trials took place. All samples were collected from animals by veterinarians as part of their routine diagnostic practices.

## 3. Results

### 3.1. Assessment of ASFV LAMP

**Figure 1:**
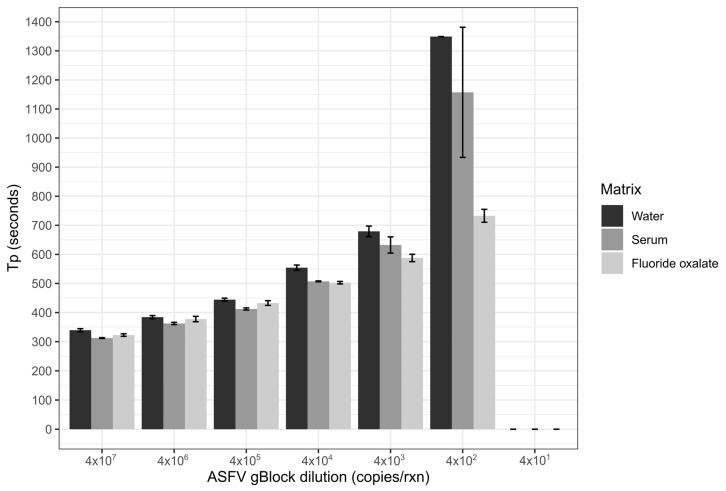
Limit of detection of the ASFV LAMP performed with the ASFV gBlock in three different sample matrices. The sample matrices of serum and fluoride oxalate blood were diluted 1 in 10 in nuclease-free water before being spiked with the ASFV gBlock. All runs were performed in triplicates with error bars representing the standard deviation.

**Figure 2:**
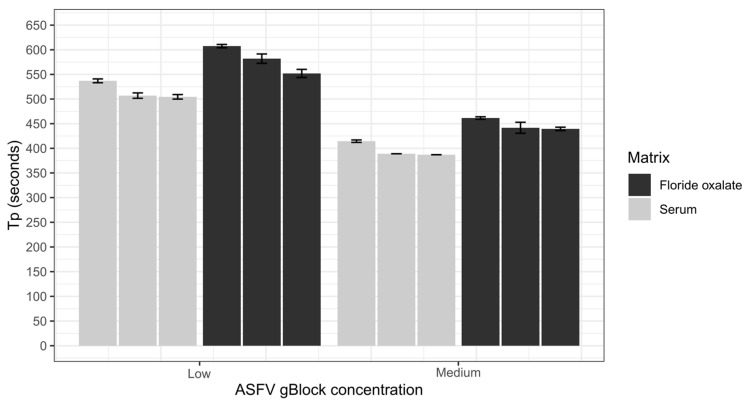
Repeatability of the ASFV LAMP assay was assessed by spiking a low (400 copies/rxn) and medium (40,000 copies/rxn) concentration of the ASFV gBlock into two sample matrices, serum and whole blood collected in fluoride oxalate tubes. All samples were tested in triplicates with error bars representing standard deviation.

The ASFV LAMP assay was successfully optimized for both primer concentration ratio and temperature. The analytical sensitivity of the assay was determined to be effectively down to 400 copies per reaction of the ASFV gBlock, when tested in the sample matrix of water, serum or fluoride oxalate blood (Figure 1). However, the fluoride oxalate matrix had a lower variability compared to the other sample matrices, with the gBlock not detected in all water replicates at this dilution (Figure 1). No detections less than 400 copies occurred for any of the sample matrices (Figure 1). Repeatability of the assay was first assessed by spiking a low and medium ASFV gBlock concentration into serum and fluoride oxalate blood (Figure 2). The coefficient of variation of T_p_ for either sample matrix or gBlock concentration was low (Table 3 and Table 4), indicating the ASFV LAMP assay had a good level of repeatability.

### 3.2. Comparison of Sample Matrices and Dilution of Anticoagulants

**Figure 3:**
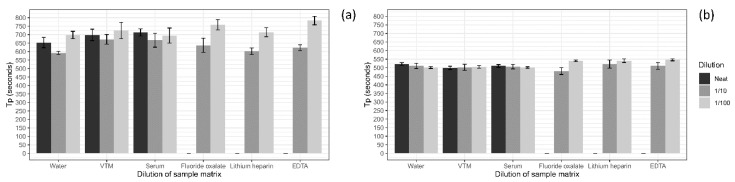
The effect of sample matrix on the detection of a low (400 copies/rxn) ASFV gBlock spike (**a**) and a medium (40,000 copies/rxn) ASFV gBlock spike (**b**), with the sample matrix tested at neat (black), 1 in 10 (dark grey) and 1 in 100 (light grey) dilution in nuclease-free water. All runs were performed in triplicates. No signal was detected for the ASFV gBlock when spiked into neat blood.

The effect of sample matrix on LAMP detection times was assessed using five different sample matrices plus water, with a low (400 copies/rxn) and medium (40,000/rxn) concentration of the ASFV gBlock. No significant difference (*p* ≥ 0.2, ANOVA) was observed between water and the undiluted serum or VTM, for either the low or medium ASFV gBlock (Figure 3). However, all blood tubes when tested undiluted solidified with the heat-treated at 95 °C for 2 min (data not shown) and hence no detections were obtained (Figure 3). Once the sample matrices were diluted 1 in 10 in nuclease-free water, there was no significant difference (*p* ≥ 0.3, ANOVA) in T_p_ for any of the sample matrix tested at either of the ASFV gBlock concentrations (Figure 3). At the 1 in 100 dilution of the sample matrix with a medium ASFV gBlock spike, a significant difference (*p* < 0.05, ANOVA) in T_p_ was observed with water, VTM and serum providing faster detection times compared to the blood sample matrices. However, when spiked with the low ASFV gBlock in the 1 in 100 dilution of the sample matrices, no significant difference in detection time was observed.

The effect of diluting the sample matrix on detection time was next assessed, with the comparison excluding the undiluted blood matrices. No difference in detection time occurred between dilutions of the sample matrix of VTM or serum at either ASFV gBlock spike. The fluoride oxalate matrix spiked with the medium ASFV gBlock had a quicker detection time at the level of significance (*p* = 0.0494, *t*-test) for the 1 in 10 dilution compared to the 1 in 100 dilution. Additionally, at the low ASFV gBlock spike a significantly quicker detection time on average of 1:52 mm:ss was observed for the 1 in 10 dilution of the lithium heparin blood (*p* = 0.02, *t*-test) and 2:40 mm:ss for the 1 in 10 dilution of the EDTA blood (*p* = 0.006, *t*-test), compared to their 1 in 100 diluted counterparts.

### 3.3. Blood Swab Assessment

**Figure 4:**
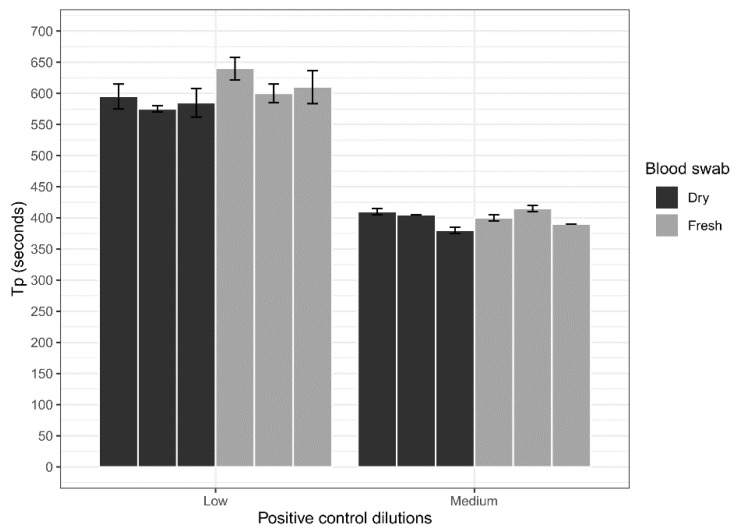
Comparison of dry and fresh blood swabs on the detection sensitivity of the ASFV LAMP assay, using porcine blood spiked with a low concentration (200 copies/µL) and medium concentration (20,000 copies/µL) of the ASFV gBlock.

To assess if dry blood swabs would affect LAMP detections a low and medium concentration of the ASFV gBlock was spiked into porcine blood. Blood swabs were successfully used to detect the ASFV gBlock. No significant difference was found comparing the dry and fresh blood swabs at the low concentration (*p* = 0.06, *t*-test) or the medium concentration (*p* = 0.61, *t*-test; Figure 4), indicating that the drying process did not affect or inhibit the ASFV LAMPs ability to detect the ASFV gBlock.

### 3.4. Analytical Specificity

To determine if there are any interactions with the unextracted sample matrix of whole blood 160 pig samples were collected and tested with the ASFV LAMP assay. None of the 160 samples produced any off-target reaction with the ASFV LAMP assay.

### 3.5. ASFV LAMP Results for Serum and Swab Samples Tested in Timor-Leste

The ASFV LAMP assay was successfully used to detect the presence of ASFV in multiple serum samples from Baucau, Timor-Leste. Of the 37 serum samples tested from this region, 11 returned a positive result (Table 5), indicating an approximate 30% occurrence of ASFV. The mean amplification time for these positive detections of ASFV in serum was seen at 9 min: 56 s (± 6 min: 10 s) with this corresponding to an annealing temperature of 87.35 °C (± 0.20 °C). A series of other samples developed a T_p_ on the ASFV LAMP assay but failed to produce the correct T_a_ (Table 5). A comparison was also performed using swabs as an alternative to serum sampling. From the 37 samples tested, 10 swab samples were positive based on ASFV LAMP (Table 6). Two serum positive samples were missed when testing swabs (Table 6, samples 13 and 20), and one sample tested positive as a swab but negative as serum (Table 6, sample 36). Of the nine samples, which tested positive by both serum and swab, there was no significant difference (*p* = 0.29, *t*-test) in T_p_. Comparison between the two sample types indicated a substantial level of agreement (k 0.798; 95% CI 0.581—1.015) based on Cohan’s kappa coefficient, when assessing the T_p_ for ASFV between the serum and swab samples.

### 3.6. Performance of Internal Amplification Control

The IAC gBlock was observed to be detected in all samples with an average detection time of 10:40 (±4:00), with an annealing temperature of 89.50 °C (±0.5 °C).

### 3.7. Cross Verification of Positives with qPCR

The sensitivity of the ASFV LAMP assay was assessed by screening all samples with the King et al. 2003, ASFV diagnostic qPCR. Samples that were processed for the qPCR were DNA extracted prior to screening. The ASFV qPCR detected 11 positive samples out of the 37 serum samples. However, two of these samples were negative by the ASFV LAMP (Table 5, samples 21 and 24), and two samples were positive by the LAMP assay but negative by the qPCR (Table 5, samples 20 and 37). The two samples that were negative by the ASFV LAMP assay had a Cq value of 35.2 and 39.1 (Table 5), with this being above the limit of detection of the ASFV LAMP assay. Comparison between the two assays indicated a substantial level of agreement (k 0.74; 95% CI 0.503—0.979) based on Cohan’s kappa coefficient, between the LAMP and qPCR results for the detection of ASFV.

### 3.8. Colourmetric LAMP

All 37 serum samples from the Baucau sample set were tested using the colourmetric assay, with thirteen samples returning a positive (pink colouration) result and two intermediate (orange colouration) results. The colourmetric LAMP assay detected 10 ASFV positives out of the 11 samples positive by qPCR. The one sample that was missed (Table 5, sample 24) by the colourmetric LAMP assay had a high Cq value, indicating that there was a low level of viral DNA in this sample. The colourmetric LAMP returned an additional two positive results that were not positive by the ASFV LAMP assay, these two samples either returned a very early T_p_ (1:40) or late T_p_ (22:55), however never developed a T_a_ for either sample.

## 4. Discussion

In recent years we have seen the continued movement and outbreak of ASFV in multiple countries, devastating the swine industry, with many of these outbreaks occurring in remote or resource-limited situations [6,12,43]. As there is no ASFV vaccine currently commercially licenced, a rapid, sensitive, specific, cost-effective test that is field ready, is needed to contain infected swine by supporting a quick diagnosis [44,45]. Numerous molecular assays have been developed for the detection of ASFV such as PCR [46], and qPCR [9]. However, these assays need expensive machines, extracted nucleic acid, and hence are primarily confined to fully established molecular laboratories. LAMP assays are perfectly positioned to fill this gap of in-field diagnostics. An ASFV LAMP assay [36] targeting the topoisomerase II gene has previously been developed to detect virus in extracted DNA with good specificity and sensitivity. We performed further development of this assay, by testing a range of unextracted sample matrices, simplifying workflow, developing an exogenous IAC to monitor for amplification inhibition, and performing in field testing of unextracted samples. Additionally, we performed a comparison of the ASFV LAMP assay, colourmetric LAMP and qPCR on a sample set in Timor-Leste.

The analytical sensitivity of the ASFV LAMP assay to detect the ASFV gBlock, when assessed in a series of sample matrices (water, serum or whole blood collected in fluoride oxalate tube), was capable of detection down to 400 DNA copies per reaction. A similar level of detection sensitivity was also observed with using both fresh and dry blood swabs (Figure 4). This is very similar to the sensitivity previously reported (330 copies of a plasmid DNA) [36]. Although, we report analytical sensitivity in a range of complex sample matrices such as serum and whole blood. Additionally, we show in a comparison between dry and fresh blood swabs that there is no loss in detection sensitivity of the ASFV gBlock or inhibition of the LAMP reaction while utilising this sampling methodology.

For the development of any molecular test, you ideally want to target the sample type that has the highest viral load; in the case of ASFV, this has been shown to be blood, followed by nasal, rectal and oral fluids [14]. As a result, an assessment of whole blood collected in a series of different anticoagulants was tested to determine if they would inhibit the LAMP reactions, as has been observed with other molecular assays such as PCR [47,48]. The presence of the anticoagulants in the samples did not hinder the detection time (T_P_) of the LAMP assay at the one in ten dilutions of these blood samples and, at times, provided quicker detections than the 1 in 100 dilution. This is consistent with other studies, which have shown a dilution of the whole blood containing the anticoagulants EDTA and heparin restores the ability of the LAMP assay to detect its target [49]. Additionally, the analytical specificity was further investigated through the testing of 160 pig bloods from an ASFV negative area, with no cross-reaction with this sample matrix detected. The assay was not assessed against other viruses, which have similar clinical presentation such as CSFV, as this had been performed in the original article [36].

Testing of ASFV clinical samples from Timor-Leste, highlighted a substantial level of agreement (k 0.74; 95% CI 0.503—0.979) based on Cohan’s kappa coefficient, between the ASFV LAMP assay and the OIE recommend ASFV qPCR. Of the 37 serum samples screened, both the LAMP and qPCR assay detected 11 positives samples. However, only nine of these samples were positive by both tests (Table 5, ‡). The ASFV LAMP missed two samples with one developing a T_p_ at 22:55 but no T_a_ (Table 5, sample 21) and one sample not developing either a T_p_ or T_a_ (Table 5, sample 24). The two samples that were negative by the ASFV LAMP had Cq values of 35.2 and 39.1, representing a low viral load. Previous studies have shown that the ASFV qPCR has a higher sensitivity being able to detect down to 10–100 molecules compared to the ASFV LAMP, which can detect down to 330–400 copies [9,36], which may explain these false negatives. However, the validity of the sample with a Cq value of 39.1 would be questioned in a typical molecular diagnostic laboratory setting with a Cq > 40 considered as a negative result [41]. Additionally, the qPCR utilizes a 5 µL addition of template as the standard diagnostic protocol compared to the 2 µL of template added to the ASFV LAMP assay.

By contrast, two samples were positive by ASFV LAMP but negative by the ASFV qPCR. This may be due to inhibitors in the sample affecting the qPCR resulting in failed detection, with previous studies showing that LAMP reactions can handle inhibitors better than qPCR [28,49]. The performance of the in-field nucleic acid extraction kit used in Timor-Leste for ASFV detection has not been well defined. This is not the first time the ASFV LAMP assay has detected samples that were missed by the qPCR, as was seen by James et al. 2010, with the results in that study confirmed by virus isolation [36].

Multiplexing of LAMP targets can be difficult and result in a reduction in target sensitivity and specificity, as has been seen for other isothermal amplification techniques [50]. Studies have shown that an increase in the number of LAMP targets in a single reaction tube can result in a slight increase in detection times [51]. The IAC utilized in this study, which takes advantage of the same primer set as the ASFV assay, was performed in separate reaction tubes to ensure no competition between the IAC gBlock and ASFV detection occurred. The IAC gBlock was successfully amplified and seen to have a T_a_ at on average 2 °C higher than that of an ASFV positive or the ASFV gBlock. With the removal of the need to perform DNA extractions, the addition of an IAC gBlock during screening can allow the confirmation of true ASFV negative samples, as opposed to an unsuccessful reaction due to inhibition [34,52].

The colourmetric LAMP assay was seen to have similar performance (11 ASFV LAMP vs. 13 colourmetric LAMP positives) to the ASFV LAMP assay. However, additional false positives were seen in the colourmetric assay, and the development of an intermediate colouration, making interpretation difficult. This occurrence of false-positives from in-direct detection methods such as, colourmetric LAMP or the use of intercalating dyes has been observed before, due to non-specific primer interactions facilitating the concatemeric LAMP products [53,54]. Although an increased chance of obtaining a false positive was observed with the colourmetric LAMP assay, only one false negative was detected, which was at the limit of detection of the ASFV qPCR (Cq 39.1). This highlights that the colourmetric LAMP may be a useful technology to screen large amounts of samples in remote locations without the need for a real-time LAMP machine [55] with positive samples triaged to other testing technologies for confirmation.

The simplicity of collecting an appropriate sample can be a major component to ensure a successful surveillance program for a disease [56]. Guinat et al. 2014 has previously identified that the highest number of ASFV genome copies are detected in blood, followed by nasal, rectal and oral fluids. To examine if a simplified sample collection using oral/rectal swabs had comparable ASFV detections to serum, 37 pigs were sampled by both means. Two additional positives were detected by serum sampling compared to swabs, with one of these samples occurring at a relatively late detection time (T_p_ = 16:10), indicating a lower level of ASFV DNA. Additionally, samples that were positive for both serum and swab had a substantial level of agreement and no significant difference (*p* = 0.29) in ASFV detection time (T_p_). The successful detection of ASFV in oral/rectal fluids via the LAMP assay also suggests that this testing methodology could be paired with more sentinel/passive surveillance, which is an area needing further investigation in ASFV surveillance [45]. Surveillance using passive chew rope detection methods for ASFV and other diseases, may be facilitated with LAMP diagnostics, especially with some studies highlighting there is currently a lack of suitable diagnostic protocols that could perform this testing [57,58].

The purpose of this study was to assess the field applicability of an ASFV LAMP assay, by removing the need to use extracted samples, the development of an exogenous IAC and perform cross-verification of the ASFV LAMP assay with an OIE recommend ASFV qPCR using clinical samples in Timor-Leste. A substantial level of agreement was seen between the LAMP assay, which used unextracted serum samples, compared to the DNA extracted samples tested on the qPCR assay. The successfully implemented IAC further provided confidence in using unextracted samples, verifying a negative sample, rather than an inhibited sample. Assessment of different sample matrices revealed that a 1 in 10 dilution in sterile water negated the effect of anticoagulants used during blood collection, resulting in no reduction in assay detection time. Although a colourmetric assay tested was observed to have an increased amount of indeterminate result, it still showed the value in an assay that could be utilized in a resource-limited situation. Furthermore, a simplified sampling means through utilization of swabs showed promise as an alternative to more difficult to acquire blood samples, with a substantial level of agreement observed between these two sampling mediums. In this study we highlighted the reliability and field applicability of the ASFV LAMP assay in the face of an ASFV outbreak in Timor-Leste; however further work needs to be conducted by increasing sample validation on clinical samples in-field before this assay can be rolled out to its full extent for use in routine in-field diagnostics.

## Figures and Tables

**Table 1:** Primer sequences used for the qPCR and loop-mediated isothermal amplification (LAMP) assays.

Assay	Target	Primer	Sequence (5′ > 3′)	Reference
qPCR	3′-end of the VP72 gene	ASF forward	CTGCTCATGGTATCAATCTTATCGA	[9]
		ASF reverse	GATACCACAAGATCRGCCGT	
		ASF probe	FAM-CCACGGGAG/Zen/GAATACCAACCCAGTG-IABkFQ	
LAMP	Topoisomerase II	F3 forward outer	GGCGCAAAATTTTAGCCGG	[36]
		B3 reverse outer	GCCGAAGCTTCCTATGCC	
		FIP forward inner	GCAACGTAGCCCCCGAACTGGAAATGCTTCGCYTCCAACA	
		BIP reverse inner	ATCACCATGGCGACATGTCGTGGATAGAGGTGGGAGGAGC	
		FLoop forward loop	AAAAACCTTTCGTTCACGGT	
		Bloop reverse loop	AAAAGCCGCCCAGTATTACC	

**Table 2:** Sequences of the African swine fever virus (ASFV) synthetic gBlock TPII and internal amplification control (IAC). The underlined section in the IAC gBlock is the altered region resulting in an elevation of the T_a_.

Synthetic DNA Control	Sequence 5′ > 3′
ASFV gBlock TPII	GGCGCAAAATTTTAGCCGGGGGGTTGAAATGCTTCGCCTCCAACAACCGTGAACGAAAGG TTTTTCAGTTTGGGGGCTACGTTGCGGATCACATGTTTTATCACCATGGCGATATGTCGTTA AACACAAGTATTATAAAAGCCGCCCAGTATTACCCAGGCTCCTCTCACCTCTATCCAGTA TTCATAGGCATAGGAAGCTTCGGC
ASFV gBlock IAC	GGCGCAAAATTTTAGCCGGGGGGTTGAAATGCTTCGCCTCCAACAACCGTGAACGAAAGG TTTTTCAGTTTGGGGGCTACGTTGCGCGACGTCACCGACGTGCCGTGATCACCATGGCGATA TGTCGTTAAACACAAGTATTATAAAAGCCGCCCAGTATTACCCAGGCTCCTCTCACCTCTAT CCAGTATTCATAGGCATAGGAAGCTTCGGC

**Table 3:** Assessment of intra-assay variation of ASFV LAMP and coefficient of variation.

Treatment	Replicate	Mean T_p_ (sec) (*n* = 6)	±SD (sec)	CV (%)
Low ASFV gBlock in fluoride oxalate blood	1	582	21	3.64
	2	608	8	1.35
	3	552	18	3.33
Low ASFV gBlock in serum	1	537	9	1.77
	2	507	13	2.65
	3	505	11	2.24
Medium ASFV gBlock in fluoride oxalate blood	1	462	6	1.33
	2	442	27	6.20
	3	440	8	1.87
Medium ASFV gBlock in serum	1	415	6	1.48
	2	389	0	0.00
	3	387	0	0.00

**Table 4:** Assessment of inter-run variation of ASFV LAMP assay and coefficient of variation.

Treatment	Mean T_p_ (sec) (*n* = 18)	±SD (sec)	CV (%)
Low ASFV gBlock in fluoride oxalate blood	581	28	4.79%
Low ASFV gBlock in serum	516	18	3.50%
Medium ASFV gBlock in fluoride oxalate blood	448	12	2.71%
Medium ASFV gBlock in serum	397	15	3.86%

**Table 5:** Results of 37 serum samples from Baucau, Timor-Leste, tested with the LAMP, qPCR and colourmetric LAMP assay. Grey highlighting indicates a positive for that particular assay, a double dagger (‡) beside the sample number indicates a positive for all four assays. qPCR results are shown for the sample as well as the positive control. Colourmetric results are negative—pink colouration (NEG), positive—yellow colouration (POS) or indeterminant—orange colouration (IND).

Sample	Age (Months)	Sex	ASFV LAMP		IAC LAMP		qPCR		Colourmetric
			T_p_ (mm:ss)	T_a_ (°C)	T_p_ (mm:ss)	T_a_ (°C)	Sample (Cq)	Positive (Cq)	
1	6	F	-	-	12:10	89.6	-	10.65	NEG
2	12	F	-	-	11:23	89.5	-	10.67	NEG
3	18	M	1:30	-	11:25	89.6	-	11.83	NEG
4	5	F	-	-	12:55	89.5	-	11.86	NEG
5	48	F	-	-	10:43	89.6	-	11.35	NEG
6	12	M	1:30		9:55	89.5	-	11.73	NEG
7 ‡	18	F	10:00	87.20	9:55	89.2	27.39	10.39	POS
8	6	M	1:30	-	13:23	89.4	-	10.47	IND
9	7	M	1:45	-	13:23	89.4	-	13.64	NEG
10	48	F	6:45	-	11:08	89.4	-	11.39	NEG
11	7	F	-	-	9:55	89.6	-	10.56	NEG
12	12	F	1:30	-	9:13	89.5	-	10.87	NEG
13 ‡	18	F	7:55	87.30	10:02	89.4	22.74	11.79	POS
14 ‡	7	F	12:10	87.40	13:55	89.4	25.4	11.57	POS
15	7	F	-	-	14:10	89.7	-	11.83	NEG
16	18	M	1:40	-	14:25	89.6	-	11.26	NEG
17 ‡	8	M	9:57	87.40	10:57	89.2	27.5	10.75	POS
18	7	F	-	-	11:12	89.6	-	11.24	NEG
19	8	M	-	-	12:57	89.4	-	11.73	NEG
20	8	F	16:10	87.20	10:38	89.6	-	11.63	POS
21	8	F	22:55	-	10:23	89.7	35.2	10.37	POS
22	24	F	1:30	-	10:27	89.6	-	11.53	NEG
23	24	F	-	-	9:27	89.7	-	11.73	NEG
24	12	F	-	-	9:57	89.6	39.1	11.43	NEG
25	12	F	-	-	10:25	89.6	-	10.87	NEG
26 ‡	18	F	9:25	87.34	8:55	89.7	25.4	11.4	POS
27 ‡	1	M	8:20	87.20	10:10	89.2	23.2	11.63	POS
28	24	F	1:40	-	9:55	89.6	-	11.73	POS
29	12	M	-	-	9:10	89.6	-	11.92	NEG
30	12	M	2:55	-	9:10	89.6	-	12.67	NEG
31 ‡	18	F	7:38	87.54	8:53	89.1	25.58	11.12	POS
32	12	M	-	-	9:38	89.7	-	12.73	NEG
33	12	M	-	-	8:38	89.7	-	11.34	IND
34 ‡	24	F	8:57	87.4	8:27	89.8	24.23	11.4	POS
35 ‡	12	M	8:45	87.53	8:42	89.8	21.2	11.73	POS
36	6	M	-	-	9:27	89.7	-	11.64	NEG
37	24	F	10:08	87.30	9:00	89.1	-	11.37	POS

**Table 6:** Results of 37 serum and matching oral/rectal swabs tested with the ASFV LAMP. Grey highlighting indicates a positive for that particular assay.

Sample	Age (Months)	Sex	Serum ASFV LAMP		Swab ASFV LAMP	
			T_p_ (mm:ss)	T_a_ (°C)	T_p_ (mm:ss)	T_a_ (°C)
1	6	F	-	-	-	-
2	12	F	-	-	-	-
3	18	M	1:30	-	18:30	-
4	5	F	-	-	-	-
5	48	F	-	-	-	-
6	12	M	1:30		-	-
7	18	F	10:00	87.2	9:53	87.58
8	6	M	1:30	-	-	-
9	7	M	1:45	-	-	-
10	48	F	6:45	-	-	-
11	7	F	-	-	-	-
12	12	F	1:30	-	-	-
13	18	F	7:55	87.3	-	-
14	7	F	12:10	87.4	14:00	87.6
15	7	F	-	-	-	-
16	18	M	1:40	-	18:00	
17	8	M	9:57	87.4	17:00	87.6
18	7	F	-	-	-	-
19	8	M	-	-	-	-
20	8	F	16:10	87.2	-	-
21	8	F	22:55	-	-	-
22	24	F	1:30	-	-	-
23	24	F	-	-	-	-
24	12	F	-	-	20:00	89.1
25	12	F	-	-	-	-
26	18	F	9:25	87.34	8:15	87.62
27	1	M	8:20	87.2	7:30	87.54
28	24	F	1:40	-	-	-
29	12	M	-	-	-	-
30	12	M	2:55	-	-	-
31	18	F	7:38	87.54	8:15	87.5
32	12	M	-	-	-	-
33	12	M	-	-	-	-
34	24	F	8:57	87.4	10:45	87.49
35	12	M	8:45	87.53	11:00	87.54
36	6	M	-	-	12:30	87.47
37	24	F	10:08	87.3	9:30	87.47
